# Sensitive and Specific Detection of the Non-Human Sialic Acid *N*-Glycolylneuraminic Acid In Human Tissues and Biotherapeutic Products

**DOI:** 10.1371/journal.pone.0004241

**Published:** 2009-01-21

**Authors:** Sandra L. Diaz, Vered Padler-Karavani, Darius Ghaderi, Nancy Hurtado-Ziola, Hai Yu, Xi Chen, Els C. M. Brinkman-Van der Linden, Ajit Varki, Nissi M. Varki

**Affiliations:** 1 Departments of Medicine, Pathology and Cellular & Molecular Medicine, Glycobiology Research and Training Center, School of Medicine, University of California San Diego, La Jolla, California, United States of America; 2 Gc-Free, Inc., La Jolla, California, United States of America; 3 Department of Chemistry, University of California Davis, Davis, California, United States of America; 4 Crucell Holland BV, Leiden, The Netherlands; Louisiana State University, United States of America

## Abstract

**Background:**

Humans are genetically defective in synthesizing the common mammalian sialic acid *N*-glycolylneuraminic acid (Neu5Gc), but can metabolically incorporate it from dietary sources (particularly red meat and milk) into glycoproteins and glycolipids of human tumors, fetuses and some normal tissues. Metabolic incorporation of Neu5Gc from animal-derived cells and medium components also results in variable contamination of molecules and cells intended for human therapies. These Neu5Gc-incorporation phenomena are practically significant, because normal humans can have high levels of circulating anti-Neu5Gc antibodies. Thus, there is need for the sensitive and specific detection of Neu5Gc in human tissues and biotherapeutic products. Unlike monoclonal antibodies that recognize Neu5Gc only in the context of underlying structures, chicken immunoglobulin Y (IgY) polyclonal antibodies can recognize Neu5Gc in broader contexts. However, prior preparations of such antibodies (including our own) suffered from some non-specificity, as well as some cross-reactivity with the human sialic acid *N*-acetylneuraminic acid (Neu5Ac).

**Methodology/Principal Findings:**

We have developed a novel affinity method utilizing sequential columns of immobilized human and chimpanzee serum sialoglycoproteins, followed by specific elution from the latter column by free Neu5Gc. The resulting mono-specific antibody shows no staining in tissues or cells from mice with a human-like defect in Neu5Gc production. It allows sensitive and specific detection of Neu5Gc in all underlying glycan structural contexts studied, and is applicable to immunohistochemical, enzyme-linked immunosorbent assay (ELISA), Western blot and flow cytometry analyses. Non-immune chicken IgY is used as a reliable negative control. We show that these approaches allow sensitive detection of Neu5Gc in human tissue samples and in some biotherapeutic products, and finally show an example of how Neu5Gc might be eliminated from such products, by using a human cell line grown under defined conditions.

**Conclusions:**

We report a reliable antibody-based method for highly sensitive and specific detection of the non-human sialic acid Neu5Gc in human tissues and biotherapeutic products that has not been previously described.

## Introduction

Humans cannot synthesize the common mammalian sialic acid *N*-glycolylneuraminic acid (Neu5Gc), due to an irreversible mutation in the gene encoding Cytidine monophosphate (CMP)-*N*-acetylneuraminic acid (Neu5Ac) Hydroxylase (CMAH) - the enzyme responsible for conversion of CMP-Neu5Gc from CMP-Neu5Ac. Thus, in comparison to our closest evolutionary relatives such as chimpanzees and gorillas, human blood cells and serum proteins lack Neu5Gc and accumulate an excess of the precursor sialic acid Neu5Ac [1], [2]. In keeping with this, normal humans have variable and sometime very high amounts of circulating antibodies directed against Neu5Gc [3]–[5]. Despite this, small amounts of Neu5Gc have been found in cultured human cells (including human embryonic stem cells) [6], and in certain tissue samples from humans [4], [7]. Larger amounts of Neu5Gc were also reported earlier in human malignant tumors and in fetal tissues (reviewed in refs. 2,4,8), and we have recently confirmed the presence of 1–3% Neu5Gc in the glycans of some human tumors [9]. Likewise, Neu5Gc has been detected in some biotherapeutic products derived from animal cells and/or grown in animal serum-containing media [10]–[18]. These findings were typically made using monoclonal or polyclonal anti-Neu5Gc antibodies, and have been confirmed by chemical analysis and mass spectrometry [4]. While mass spectrometric analysis is more definitive, it is not practical for routine laboratory use, nor is it sensitive enough in some situations. Furthermore, mass spectrometry lacks the ability to carry out detection in situ, on cell surfaces or on tissue sections.

On the other hand, the anti-Neu5Gc antibodies used to date also have limitations. Monoclonal antibodies can be highly specific for certain Neu5Gc containing glycans, but do not have the ability to detect Neu5Gc on other structurally-related or unrelated glycans [19], [20]. Thus, polyclonal antibodies raised in chickens (another species that make an immune response to Neu5Gc) have been traditionally used to detect Neu5Gc [21]–[30]. In an attempt to improve specificity and reduce background reactivity, such antibodies have been affinity-purified from whole egg IgY from immunized chickens [21]–[23], [25]. A few years ago, we made an attempt to further improve the specificity of such antibodies by additional modifications of the affinity procedures [4]. However, even with such improved preparations we noted a small amount of cross-reactivity with non-Neu5Gc-containing glycans. This was particularly clear when we did immunohistochemical studies of embryos and tissues from mice with an induced human-like genetic defect in Neu5Gc production [31]. Despite the complete absence of Neu5Gc in mass-spectrometric analyses in this well-defined situation, we still saw some background reactivity in some of these mouse tissues. The likely explanation for this appears to be a low level of cross-reactivity with high densities of other sialic acids, such as the human sialic acid *N*-acetylneuraminic acid (Neu5Ac). Thus, there remains a need for a highly sensitive and specific method to routinely detect Neu5Gc in all its presentation forms, in human tissues and cells, as well as in biotherapeutic products intended for human use. Here we remedy this deficiency using a new approach that allows highly sensitive and specific detection of Neu5Gc with applications in a variety of methodologies.

## Materials and Methods

### Chemicals, Reagents and Buffers

The sources of some reagents are listed within the method descriptions below. The following materials were from the sources indicated: Affi-Gel 15, Dowex AG50WX-2 and Dowex AG1X8, Bio-Rad, Richmond, CA; *N*-glycolylneuraminic acid (Neu5Gc) Inalco Pharmaceuticals, San Luis Obispo, CA; ExCell™ VPRO medium, JRH biosciences; Isogel agarose isoelectric focusing (IEF) plates, pH 3–10, Cambrex; Isoelectric Focusing Marker, Sigma; Ureum, USB; Servalyt 3–7 ampholytes, Serva; 1,2-Diamino-4,5-methylene-dioxybenzene (DMB), Cold Water Fish Skin Gelatin, Tween-20, Sigma Chemical Co., St. Louis, MO, Sigma-Aldrich; GlykoSep R high performance liquid chromatography (HPLC) column, Glyko; Sialic Acid Reference panel, Glyko; Quantikine®IVD® Erythropoietin ELISA from R&D Systems Inc. Buffers used were: Tris Buffered Saline (TBS) was prepared with 50 mM Tris HCl, 150 mM NaCl, pH 8; TBST, TBS containing 0.1% Tween-20; Phosphate buffered saline (PBS). Pairs of defined glycan structures containing Neu5Ac or Neu5Gc attached to polyacrylamide (PAA) were from Glycotech, and those attached to human serum albumin (HSA) were prepared as previously described [32].

### Initial Preparation of Crude Chicken Polyclonal Antibody Against Neu5Gc

Ganglioside Neu5Gcα2-3Galβ1-4G1cβ1-1'Ceramide (GM_3_(Neu5Gc)) was prepared from horse erythrocytes [33] and used to immunize 2 Rhode Island Red chickens with 1 mg immunogen in Freund's complete Adjuvant containing 1 mg bovine serum albumin (BSA), followed with boosts of 50 µg ganglioside in incomplete Freund's Adjuvant every three weeks. Eggs were collected and the yolks kept at 4°C until used. Total IgY was purified from the egg yolk by precipitating to 50% saturation with ammonium sulfate. The pellet was suspended in PBS, and dialyzed against PBS using 10,000 Molecular weight cut-off (MWCO) tubing. The crude IgY prep was stored frozen until affinity purification.

### Preparation of Pre-clearing and Affinity Purification Columns

The pre-clearing column was prepared by coupling pooled human type AB serum sialoglycoproteins (PelFreez, Rogers, AR) to Affigel-15 activated immunoaffinity support according to the manufacturer's instructions. The affinity column was prepared by similarly coupling chimpanzee serum (obtained from Yerkes National Primate Research Center) to Affi-Gel 15. For each column, 10 ml of resin was washed with 30 ml of 50 mM Sodium acetate pH 5.5, mixed with one ml of serum diluted to 14 ml of 100 mM 3-(*N*-morpholino)propanesulfonic acid (MOPS) pH 7.5, and gently agitated overnight on an end-over-end mixer at 4°C. Any remaining active esters were blocked by the addition of 1 ml of 1 M Ethanolamine and continued mixing for one hour. The resin was poured into a glass column and washed with 3 column volumes of 50 mM Sodium acetate pH 5.5. The sialic acid content of bound glycoproteins was determined by performing mild acid hydrolysis (0.18 N H_2_SO_4_ for 1 hour at 80°C followed by neutralization with NaOH) on an aliquot of each resin, and by performing the thiobarbituric acid (TBA) assay on the supernatants containing the released sialic acids.

### Pre-clearing of the IgY pool and Isolation of the anti-Neu5Gc specific antibodies

The crude immune IgY preparation was applied to the human serum Affi-Gel 15 pre-clearing column and allowed to run through. The effluent was collected and reapplied to the same column to allow for maximum binding of any non-specific or Neu5Ac-recognizing IgY antibodies to the Neu5Ac-containing human serum glycoproteins. The column was then washed with 10 ml of PBS, pH 7.4 and the washes combined with the column effluent. The pooled washes were applied onto the chimpanzee serum affinity column, the effluent collected and reapplied to the same column to allow for maximum binding of anti-Neu5Gc antibodies. The column was washed with 50 ml of PBS, pH 7.4, and the specific antibody then eluted from the resin using 5 mM Neu5Gc in PBS, pH 7.4. The first 5 ml of the eluting buffer was added to the column and allowed to run through. The column was then closed off, a second 5 ml of the eluant added (to prevent the column from drying out) and allowed to stand overnight, for a more complete elution of the antibody from the column. The following day, 11 additional 5 ml fractions were collected, by washing with the same eluant. The Neu5Gc-reactive fractions were detected by ELISA, using Costar high-binding plates coated in alternating rows with either 2.5 µg per ml of Neu5Gc or Neu5Acα-PAA-Biotin (Glycotech) or with 1 µl of Bovine or Human serum in 100 µl of 50 mM sodium carbonate-bicarbonate buffer, pH 9.5 at 4°C overnight. The wells were washed with TBS pH 7.5 followed by blocking with 200 µl of TBST at room temperature for 1 hr. For the identification of anti-Neu5Gc positive fractions, 100 µl aliquots of dilutions of the eluted affinity column fractions were added to each well and incubated at room temp for 2 hr. The wells were washed with TBS pH 7.5 followed by the addition of 100 µl of a 1∶10,000 dilution of Donkey anti-chicken IgY-horseradish peroxidase (HRP) (Jackson ImmunoResearch) in TBST and incubated at room temp for 1 hr. The wells were washed and developed using 140 µl of a developing solution (20 ml of citrate phosphate buffer, pH 5.5, 10 mg of *O*-phenylenediamine (OPD), 50 µl of 30% hydrogen peroxide). Once adequate color had developed, the reaction was quenched with 40 µl of 4 M Sulfuric acid, and the absorbance was read at 490 nm.

Fractions that were positive for anti-Neu5Gc antibodies were pooled and concentrated to 0.5 ml using an Amicon Ultrafree-15 10,000 molecular weight cut-off centrifugal filter device. To reduce the concentration of free Neu5Gc in antibody solution, 15 ml of PBS, pH 7.4 was added to the filtration device and the sample concentrated again to 0.5 ml. This process was repeated two more times until the concentration of the Neu5Gc was reduced to about 20 µM, a concentration too low to affect antibody binding in any studies. A typical protein concentration of the purified antibody was 5–9 mg/ml. After the elutions, both the Human serum and Chimp serum Affi-Gel 15 columns were washed with 50 ml of PBS, pH 7.4 followed by cleaning with 50 ml of 0.1 M Citric Acid pH 3.0, and immediately washed again with 50 ml of PBS, pH 7.4 to neutralize the acid. The columns were stored in PBS, pH 7.4 containing 0.5% Sodium azide for future use.

### ELISA Analysis for Neu5Gc Specificity of the Affinity-purified IgY Antibody

Anti-Neu5Gc antibody reactivity was detected by ELISA as previously described [5]. Briefly, we used 96-well microtiter plates (Costar) coated in triplicates with optimized buffers and saturating concentrations of various pairs of Neu5Ac- and Neu5Gc-glycoconjugates as follows: GM3 ganglioside in methanol (50 pmole sialic acid/well); Neu5Gc/Acα-PAA-Biotin (250 ng/well) and Neu5Gc/Ac2-3/6Lac-HSA-Biotin (1 µg/well); ovine submaxillary mucin (OSM) and porcine submaxillary mucin (PSM) (100 pmole sialic acid/well), and bovine submaxillary mucin (BSM) or base-treated BSM (190 pmole sialic acid/well) in 50 mM sodium carbonate-bicarbonate buffer, pH 9.5. Pairs of Neu5Gc/Acα2-6GalNAc-HSA-Biotin (1 µg/well), 9-OAc- Neu5Gc/Acα2-6GalNAc-HSA-Biotin (1 µg/well), in 50 mM sodium phosphate buffer, pH 7.5 (an optimal pH for preserving *O*-acetylation of sialic acid). In addition, we coated wells with Human or Bovine Fibrinogen (Sigma, St Louis, MO) (0.5 µg/well), serum from C57BL/6 wild-type or *Cmah−/−* mice, human or chimpanzee serum (diluted 1∶1,000/well). Methanol was allowed to evaporate completely for 4 hours at room temperature (RT) and plates were incubated overnight at 4°C. Wells were blocked for 1 hours at RT with 1% ovalbumin (Sigma, St Louis, MO; Grade V, free of Neu5Gc) in PBS, followed by incubation with affinity purified chicken anti-Neu5Gc diluted 1∶10,000 in the same blocking solution for 2 hours at RT. The plates were washed three times with PBS containing 0.1% Tween (PBST) and subsequently incubated for 1 hour at RT with HRP-conjugated donkey-anti-chicken IgY diluted in PBS (1∶10,000; Jackson ImmunoResearch, West Grove, PA). After washing three times with PBST, wells were developed with *O*-phenylenediamine in citrate-PO_4_ buffer, pH 5.5, and absorbance was measured at a 490 nm wavelength on a SpectraMax 250 (Molecular Devices). Neu5Gc-specific antibody levels were defined by subtracting the readings obtained with the Neu5Ac-glycoconjugates from the readings obtained using the respective Neu5Gc-glycoconjugates (in the case of naturally-occurring molecules containing Neu5Gc, the background subtracted was that of triplicate wells containing only the respective buffer).

### Western Blot Analysis

Sodium dodecyl sulfate polyacrylamide gel electrophoresis (SDS-PAGE) separated proteins were transferred to nitrocellulose membranes following standard procedures. Membranes were blocked overnight at 4°C with 0.5% gelatin from cold water fish skin (Sigma) in TBST, and then incubated at room temperature for 2 hr with affinity purified chicken anti-Neu5Gc diluted 1∶100,000 with TBST or with a control non-specific Chicken IgY antibody pool (Jackson ImmunoResearch) at the same protein concentration. The membranes were washed again with TBST and then incubated with Donkey anti-chicken HRP (Jackson ImmunoResearch) 1∶50,000 in TBST at room temperature for 1 hr. The membranes were washed and incubated with Pierce SuperSignal West Pico Substrate (Pierce) as per manufacturer's recommendation, exposed to X-ray film and the film developed.

### Flow Cytometry Analysis

The blocking solution used for all the analysis, manipulations and dilutions was 0.5% cold water fish skin gelatin in PBS, pH 7.3 containing 1 mM ethylenediaminetetraacetic acid (EDTA). Chinese hamster ovary-K1 (CHO-K1) cells were detached from the tissue culture dish using 10 mM EDTA in PBS, pH 7.3 for 5 to 10 min. The cells were immediately washed in blocking buffer containing 5 mM EDTA and counted. Peripheral blood mononuclear cells (PBMCs) were prepared by standard Ficoll-Paque Plus protocol, and washed in blocking buffer. Once prepared, 1×10^6^ cells were used for each staining. All staining reactions were performed at 4°C. The cell pellet was gently resuspended in 100 µl of either affinity purified chicken anti-Neu5Gc antibody or control pre-immune IgY diluted 1∶4000 in blocking solution and incubated on ice for 1 hr. The cells were washed with 1 ml of blocking buffer, mixed gently, and pelleted at 500×g for 5 min. The cells were suspended in 100 µl Cy5-conjugated Donkey-anti-chicken IgY antibody, diluted 1∶4000 in blocking buffer, incubated on ice for 1 hr, and washed as above. Stained cells were suspended in 400 µl PBS, the data collected on a FACSCalibur (BD Biosciences Immunocytometry Systems, San Jose, CA) and analyzed with Flowjo software (Tree Star, Ashlan, OR).

### Immunohistochemical Analysis

Frozen sections or paraffin sections of wild type mouse embryos, or wild type adult mouse organs, along with similar sections from CMAH null tissues, were used initially to confirm specificity of antibody binding to Neu5Gc containing tissues, with no binding seen to the CMAH null tissues (collection of mouse tissues from euthanized animals adhered to UCSD institutional guidelines for the ethical treatment of animals). When studying human tissues, frozen sections or paraffin sections of human placenta were always used as positive controls, because staining of endothelial cells lining blood vessels was the control necessary for interpretation of staining on other tissues. The anti-Neu5Gc antibody or Chicken IgY concentrations used on human sections were each at ∼5 micrograms per ml on frozen or on paraffin sections (1∶1000 or 1∶500 respectively - when detecting larger amounts of Neu5Gc in animal tissues it is possible to use dilutions of 1∶10,000 or 1∶20,000). The frozen sections were air-dried, and washed in phosphate buffered saline with 0.1% Tween (PBST) and overlaid with blocking buffer (0.1% fish gelatin in PBST). The washes were performed between each step and the blocking buffer was used for dilution of each of the reagents that were overlaid onto the tissue sections. The slides were incubated in a humid chamber with parafilm on them, to prevent drying during the incubation steps. The sections were blocked for endogenous biotin, overlaid with primary reagents, the binding of which was detected using a secondary biotinylated secondary, and a labeled streptavidin.

If paraffin sections were used in an immunohistochemistry assay, the slides were de-paraffinized before proceeding with the steps outlined above. Slides were immersed in 3 changes of xylene for 10 minutes each to remove the wax, followed by rehydration in decreasing concentrations of alcohol, before submersion in buffer. Following the biotinylated secondary antibody enhancement steps using the DAKO CSA kit (which uses the biotinyl tyramide amplification method) was used. We also found that following incubation with the primary reagents, specific binding could be detected using a secondary rabbit anti-chicken antibody (Jackson ImmunoResearch), followed by a fluorescently or enzyme labeled tertiary anti-rabbit antibody (Jackson ImmunoResearch). When Horse Radish Peroxidase (HRP) was the label, endogenous peroxidases were blocked before proceeding with the other steps and color was developed using peroxidase substrates (Vector labs) following manufacturer's recommendations, and nuclei were counterstained using Mayer's hematoxylin before coverslipping using aqueous mounting media. If fluorescent labels were used, the slides were mounted in aqueous mounting media and viewed using epifluorescence with a Zeiss Axiophot microscope, digital images were captured using the Scion NIH image program and analyzed using Adobe Photoshop. With the color read-out, digital images were captured on an Olympus BH2 microscope, using the Magnafire digital photocapture program.

## Results and Discussion

### The affinity purified chicken anti-Neu5Gc antibody is stable under a variety of conditions

A polyclonal monospecific chicken anti-Neu5Gc antibody was prepared as described in “Materials and Methods” by sequential application to a pre-clearing column of immobilized human serum sialoglycoproteins (glycosidically-bound Neu5Ac only, in various linkages) followed by a column of immobilized chimpanzee serum sialoglycoproteins (glycosidically-bound Neu5Ac and Neu5Gc in approximately equal amounts, in various linkages). Specific elution of the antibodies using Neu5Gc from the second column was followed by simultaneous concentration and reduction of the free Neu5Gc to insignificant levels. The activity of the final chicken anti-Neu5Gc antibody preparation was assayed for stability to pH, freeze-thaw and lyophilization, using 1 hr or 3 hr incubations at 4°C in various buffers and pH's. The activity (as determined by ELISA against Neu5Gcα-PAA) remained intact in pH ranges of 4–10 and was highest in 100 mM Sodium acetate pH 4. However a 58% loss of activity was observed when the antibody was incubated in 100 mM Citrate buffer pH 3.0 (note that prior attempts at affinity chromatography typically eluted such IgY antibodies under such low pH conditions). There was no loss of ELISA reactivity after 10 rounds of lyophilization at pH 7.3. Storage at room temperature for 3 days after lyophilization also did not appreciably reduce the activity in most assays. The antibody was also stable to extended storage at 4°C in PBS pH 7.3 for 6–8 weeks in various protein concentrations. There was an appreciable loss of activity when the protein concentration dropped below 60 µg/ml but not when the protein concentration was kept in the range of 0.6–6 mg/ml. Prolonged storage at 4°C or repeated freeze-thaw can result in some background reactivity with the control IgY, likely due to formation of aggregates. In addition, prolonged 4°C storage of the reconstituted lyophilized antibody somewhat diminished its binding capacity in immunohistochemistry.

### Affinity-purified antibody recognizes Neu5Gc with various linkages to underlying glycans

**Figure 1:**
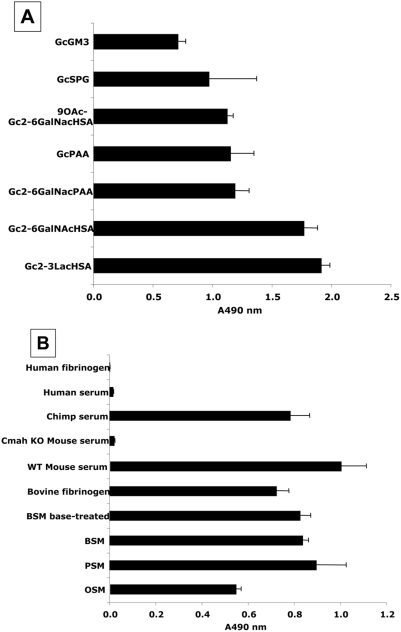
Affinity purified Chicken IgY antibody recognizes Neu5Gc with various linkages to underlying glycans. A. Detection of Neu5Gc on synthetic and semi-synthetic molecules. Reactivity of the affinity-purified anti-Neu5Gc antibody was tested in triplicate, by ELISA against synthetic and semi-synthetic Neu5Gc- and Neu5Ac-target pairs, using Neu5Ac-glycans for background subtraction (the Neu5Ac A490 value was subtracted from the corresponding Neu5Gc A490 value). B. Detection of Neu5Gc on natural glycoproteins. The affinity-purified anti-Neu5Gc antibody was tested in triplicates by ELISA, against natural glycoproteins containing Neu5Gc and Neu5Ac, using buffer only for background subtraction. Error bars indicate the standard deviation of triplicates. Gc, alpha-linked Neu5Gc; GM3, GM3 ganglioside; HSA, Human serum albumin; PAA, polyacrylamide; and, SPG, sialylparagloboside.

Sialic acids can be bound to underlying glycans by α2-3, α2-6, or α2-8 glycosidic linkages. Presumably because the binding pocket of antibodies can accommodate >4 monosaccharides [34]–[37], all mouse and chicken monoclonal Neu5Gc-dependent antibodies reported to date show specificity for both the sialic acid linkage and the precise structure of the underlying glycan chain [19], [20]. Despite the fact that the primary immunogen used to immunize chickens (GM3-Neu5Gc) contained Neu5Gc only in the α2-3linkage, the final antibody preparation reacted with all linkages and underlying glycan structures tested. A likely explanation is that the BSA that is traditionally used as a carrier is contaminated with Neu5Gc-containing bovine serum sialoglycoproteins (our unpublished observations). Regardless of the reason, this broad specificity was found both with synthetically prepared compounds (Figure 1A) and with naturally-occurring molecules containing Neu5Gc (Figure 1B). Even *O*-acetylation of the glycerol-like side chain of sialic acid, such as that found in Bovine submaxillary mucin (BSM) did not block recognition, as reactivity was not altered by prior de-*O*-acetylation of BSM by base treatment (Figure 1B). These results are typical of many ELISAs done with Neu5Gc-containing molecules. We have so far not encountered any instance where the antibody did not detect such molecules.

In every case tested, there was little or no reactivity with the Neu5Ac-containing counterpart (data not shown), confirming the extreme specificity of the antibody for the single oxygen atom that differs between Neu5Gc and Neu5Ac. This contrasts with prior methods of preparing chicken polyclonal anti-neu5Gc antibodies (including ours), which showed varying degrees of cross-reactivity with Neu5Ac-containing epitopes, as well as other non-specific reactivity. This improvement likely resulted from both the pre-clearing step using immobilized human serum sialoglycoproteins (containing only Neu5Ac), and from the specific elution by Neu5Gc from the Neu5Gc-containing immobilized chimpanzee serum sialoglycoproteins. The pre-clearing using human proteins also helped to eliminate other non-specific cross-reactivities when studying human tissue samples. To further ensure specificity, all subsequent studies used a commercial pooled batch of IgY as a control, which showed no anti-Neu5Gc reactivity.

### Sensitive and specific detection of Neu5Gc on glycoproteins by Western blotting

**Figure 2:**
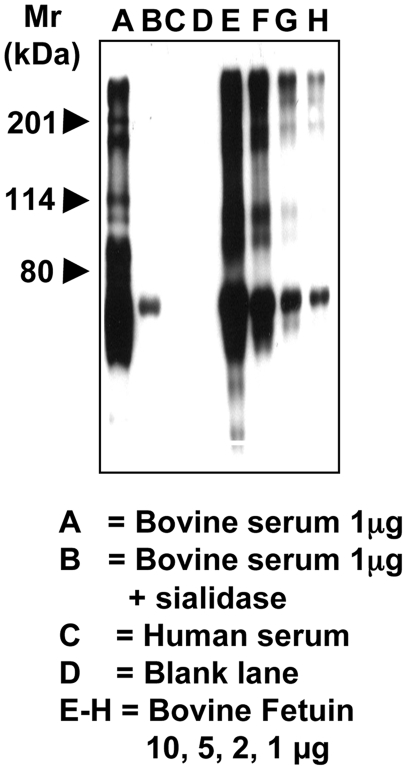
Sensitive and specific detection of Neu5Gc-containing glycoproteins by Western Blot Analysis. Standard proteins were run on a 10% SDS-PAGE gel and transferred onto nitrocellulose membrane. The membrane was blocked overnight at 4°C with Neu5Gc-free 0.5% gelatin from cold water fish skin (Sigma) in TBST. The membranes were incubated at room temperature for 2 hr with the affinity-purified chicken anti-Neu5Gc diluted 1∶100,000 with TBST. The membranes were washed with TBST and then incubated with Donkey anti-chicken HRP (Jackson ImmunoResearch) 1∶50,000 in TBST at room temperature for 1 hr. The membranes were washed and incubated with Pierce Super-Signal West Pico Substrate (Pierce) as per manufacturer's recommendation, exposed to X-ray film and the film developed. A, bovine serum 1 µg; B, bovine serum 1 µg+sialidase; C, human serum; D, blank lane. E–H, bovine fetuin 10, 5, 2, 1 µg. The smallest amount of bovine fetuin contained only 4 pmoles of Neu5Gc per µg of protein as determined by DMB-HPLC.

Human serum and bovine serum proteins were electrophoresed on 10% SDS-PAGE gels and transferred onto nitrocellulose membranes as described in the Methods. While human serum did not react at all with the antibody, the lane for bovine serum shows a full spectrum of positive bands (Figure 2, lane A). Sialidase pretreatment of the bovine serum abolished almost all reactivity confirming the specificity for sialic acids (Figure 2, lane B). There was no background staining seen when probing the blot with the control IgY antibody, nor with secondary antibody alone (data not shown). Thus, this antibody preparation can detect a complex mixture of Neu5Gc-containing proteins in a specific manner. Bovine fetuin was also used for a sensitivity check, as it carries a variety of different sialic acid linkages and but only a low amount of Neu5Gc (∼2% of total sialic acids). Low µg amounts of fetuin and asialofetuin were electrophoresed into a 10% SDS-PAGE gel. Only the fetuin gave positive reactions, giving detection of as little as 4 pmoles Neu5Gc contained in 1 µg of protein (Figure 2). This is more sensitive than conventional detection of Neu5Gc by DMB-derivatization and HPLC analysis with fluorescent detection [38] (previously the most sensitive method, with a detection limit of ∼10–20 pmoles, depending on the background).

### Sensitive and specific detection of Neu5Gc on cell surfaces by flow cytometry

**Figure 3:**
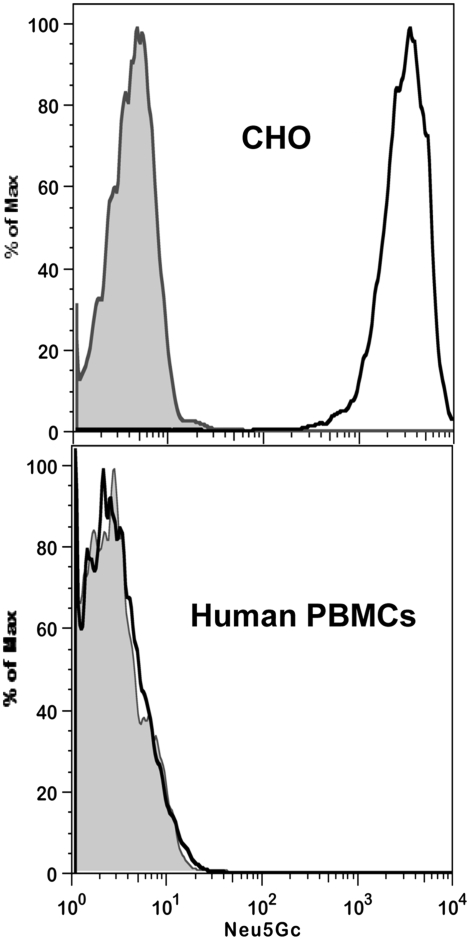
Sensitive and specific detection of Neu5Gc on cells by flow cytometry. CHO-K1 cells were detached from the tissue culture dish using 10 mM EDTA in PBS, pH 7.3. The cells were immediately washed in blocking buffer (0.5% gelatin from cold water fish skin in PBS, pH 7.5) containing 5 mM EDTA and counted by hemocytometer. Peripheral blood mononuclear cells (PBMCs) were isolated with Ficoll Paque Plus (GE Healthcare), washed in blocking buffer and counted. 1×10^6^ cells were used for each staining which was done at 4°C. The cells were washed with 1 ml cold blocking buffer then pelleted at 500×g for 5 min. The supernatant was carefully removed and discarded. The cell pellet was gently suspended in 100 µl of either affinity purified chicken anti-Neu5Gc antibody or control non-specific IgY antibody diluted 1∶4000 in blocking solution and incubated on ice for 1 hr. The cells were washed by adding 1 ml of blocking buffer, mixed gently, and pelleted as above. The cell pellet was gently suspended in 100 µl Donkey-anti-chicken IgY Cy5-conjugated diluted 1∶4000 in blocking buffer and incubated on ice for 1 hr. The cells were washed as above, resuspended in 400 µl PBS, analyzed on a FACSCalibur (BD Biosciences Immunocytometry Systems, San Jose, CA) and the data analyzed using Flowjo software (Tree Star, Ashlan, OR). Human PBMCs were negative for Gc (bottom), while mouse PBMCs (data not shown) and CHO cells were positive for Gc (top). The gray peak represents cells stained with total IgY of un-immunized chickens, and the black trace represents cells stained with anti-Neu5Gc.

Flow cytometry of various cell types was carried out as described in Methods. While human PBMCs, which do not express Neu5Gc gave no significant signal, CHO cells which express only 2–3% Neu5Gc gave a clear positive signal (Figure 3). Mouse PBMCs, which express higher levels of Neu5Gc gave the highest signal (data not shown.) None of these cells had any background staining when incubated with the control IgY antibody, nor with secondary antibody alone (data not shown). Thus the antibody is sensitive and specific for Neu5Gc in flow cytometry analysis and is capable of picking up very low levels of cell surface Neu5Gc.

### Sensitive and specific detection of Neu5Gc by immunohistochemistry in mouse and human tissues

**Figure 4:**
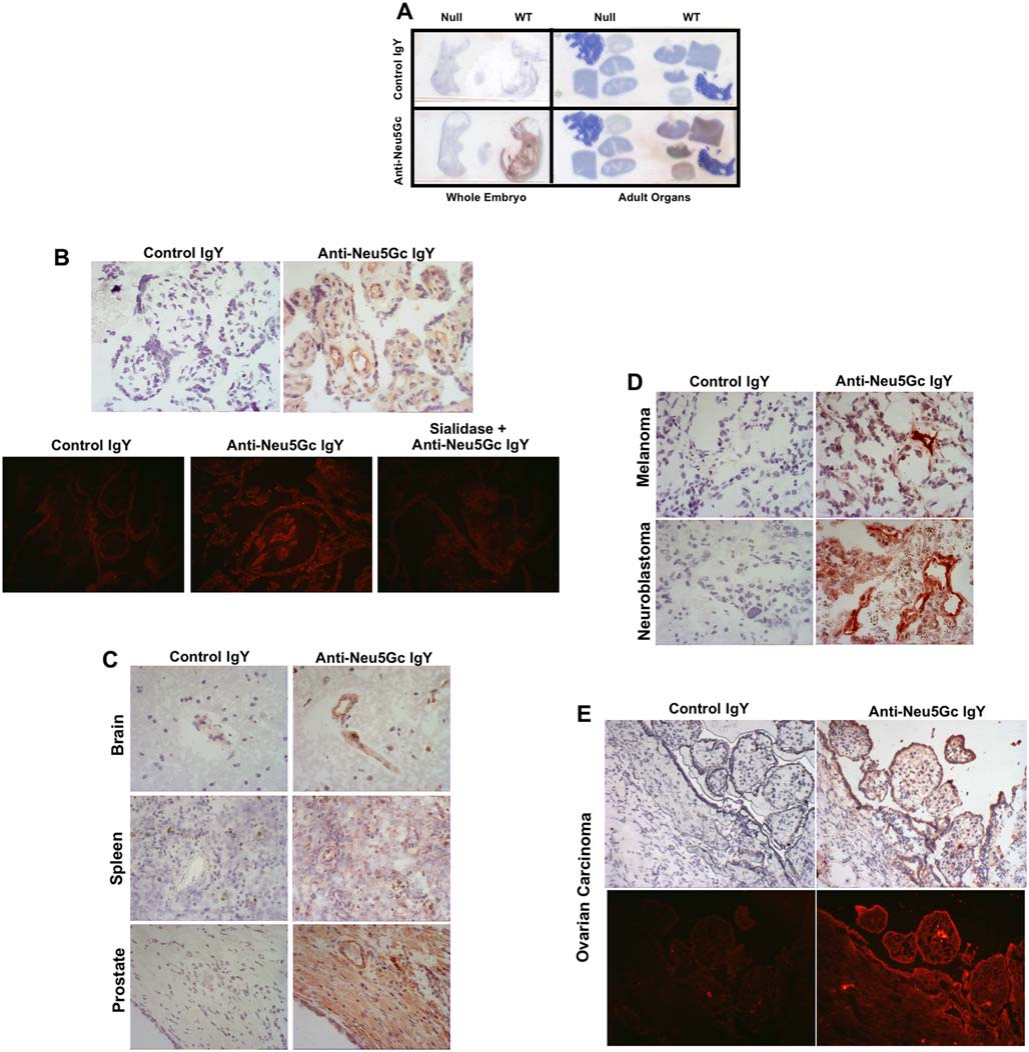
Sensitive and specific detection of Neu5Gc in mouse and human tissues by immunohistochemistry. A. Direct scan of glass slides with frozen sections of mouse embryos d16.5 or paraffin sections of adult mouse tissues, and detected using biotinylated anti-chicken antibody followed by HRP-Streptavidin. B. Frozen sections of Human Placenta immunostained with primary reagents at 1∶1000 each (5 ug/ml each) and detected using biotinylated anti-chicken antibody, followed by HRP-Streptavidin (top) or CY3-Streptavidin (bottom). (400× magnification) C. Frozen sections of normal human tissues immunostained with primary reagents at 1∶1000 each (5 ug/ml each) and detected using biotinylated anti-chicken antibody, followed by HRP-Streptavidin. (400× magnification) D. Frozen sections of examples of human tumors immunostained with primary reagents at 1∶1000 each (5 ug/ml each) and detected using biotinylated anti-chicken antibody, followed by HRP-Streptavidin (400× magnification). E. Frozen sections of human ovarian carcinoma immunostained with primary reagents at 1∶1000 each (5 ug/ml each) and detected using biotinylated anti-chicken antibody, followed by HRP-Streptavidin (top) or CY3-Streptavidin (bottom) (200× magnification).

Immunohistochemistry studies of various human and mouse tissue sections were carried out as described in Methods, with the control IgY in comparison. A few examples of the results are shown in Figure 4. Whole embryos and multiple adult organs from wild-type mice showed strong staining, whereas those from *Cmah*-null mice showed no staining (Figure 4A). This improves on our earlier work, which used a version of the chicken polyclonal antibody that was not as rigorously purified, which showed some non-specific reactivity with the *Cmah*-null mouse tissues [31]. Studies of normal human placenta showed easily detectable staining within the endothelia of blood vessels (4B). Most normal human tissues studied using frozen or paraffin sections, consistently showed staining of the blood vessels, and sometimes also of the glandular epithelial cells of breast, luminal edge of colonic mucosal epithelial cells, crypt epithelium of small intestine, some glandular epithelium of prostate, kidney glomeruli and interstitial capillaries and lung bronchial epithelium (some examples are shown in Figure 4C). Many malignant tumors and the angiogenic blood vessels also showed staining with the anti-Neu5Gc antibody (breast carcinoma, prostate carcinoma, ovarian carcinoma), while some like melanoma and neuroblastoma, only showed staining of blood vessels. In general, staining of paraffin-embedded sections of the same samples showed primarily staining of blood vessels and not much else, likely due to the extraction of glycolipids during the embedding procedure. Staining could also be enhanced using a rabbit anti-chicken antibody followed by a labeled anti-rabbit antibody (both from Jackson ImmunoResearch) if needed. In no case did any of the tissue sections show obvious background staining when incubated with the control IgY antibody, nor with secondary antibody alone. Thus the new antibody is sensitive and specific for Neu5Gc in immunohistochemical analyses. Additional examples can be seen in a recent study from our lab, where we used the antibody to confirm the presence of Neu5Gc on normal human colon and kidney samples [7].

### Detection of Neu5Gc in some FDA-approved biotherapeutic antibodies

**Figure 5:**
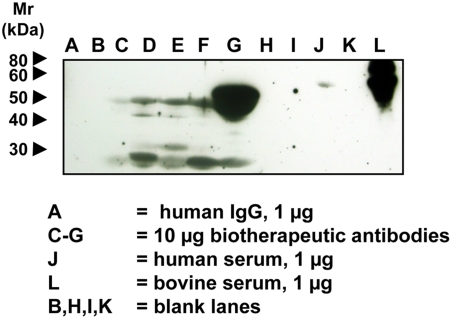
Detection of Neu5Gc on Some FDA-Approved Biotherapeutic Antibodies. Biotherapeutic agents, human IgG (Jackson Immunoresearch), human serum and bovine serum samples were run on a 12.5% SDS-PAGE gel and transferred onto a nitrocellulose membrane. The membrane was blocked overnight at 4°C with 0.5% gelatin from cold water fish skin (Sigma) in TBST. The membranes were incubated at room temperature for 2 hr with the affinity-purified chicken anti-Neu5Gc diluted 1∶100,000 in TBST with 0.5% gelatin from cold water fish skin or with a control non-specific Chicken IgY antibody pool (Jackson ImmunoResearch) at the same protein concentration. The membranes were washed with TBST and then incubated with Donkey anti-chicken HRP (Jackson ImmunoResearch) 1∶50,000 in TBST with 0.5% gelatin from cold water fish skin and 1% human serum at room temperature for 1 hr. The membranes were washed again and incubated with Pierce SuperSignal West Pico Substrate (Pierce) as per manufacturer's recommendation, exposed to X-ray film and the film developed. A, human IgG 1 µg; B, blank lane; C–G, 10 µg each of various FDA-approved biotherapeutic IgG molecules; H and I, blank lanes; J, human serum, 1 µg; K, blank lane; L, bovine serum, 1 µg.

Biotherapeutic monoclonal antibodies were electrophoresed on 12.5% SDS-PAGE gels and transferred onto nitrocellulose membranes as described in the Methods. Representatives of biotherapeutic monoclonal antibodies studied were all IgG1 molecules: either chimeric or humanized mouse monoclonal antibodies. There was no background staining seen in the blot neither with the control IgY antibody, nor with secondary antibody alone (data not shown). The anti-Neu5Gc antibody gave staining on all these glycoprotein biotherapeutic agents (Figure 5, lane C–G) most likely representing Neu5Gc originating from the mammalian expression system used and/or animal-derived serum or growth medium additives. Human IgG antibodies and human and bovine serum were used as controls (Figure 5; A, J, L). Interestingly, even some human sera gave a faint band, which might be explained by incorporation of exogenous Neu5Gc into a specific human serum glycoprotein.

The content of Neu5Gc in the biotherapeutic agents was also measured by DMB-derivatization and HPLC analysis with fluorescent detection, and the results were found to range from undetectable levels up to 0.6 pmole of Neu5Gc per microgram protein in the highest instance. Thus, using this anti-Neu5Gc IgY antibody preparation in Western blotting appears to be more sensitive than DMB-derivatization followed by HPLC fluorescent analysis, and is likely detecting even smaller amounts of Neu5Gc on FDA-approved biotherapeutic agents.

### Lack of Neu5Gc in a biotherapeutic agent prepared in the human PER.C6® cell line under Neu5Gc-free/serum-free conditions

**Figure 6:**
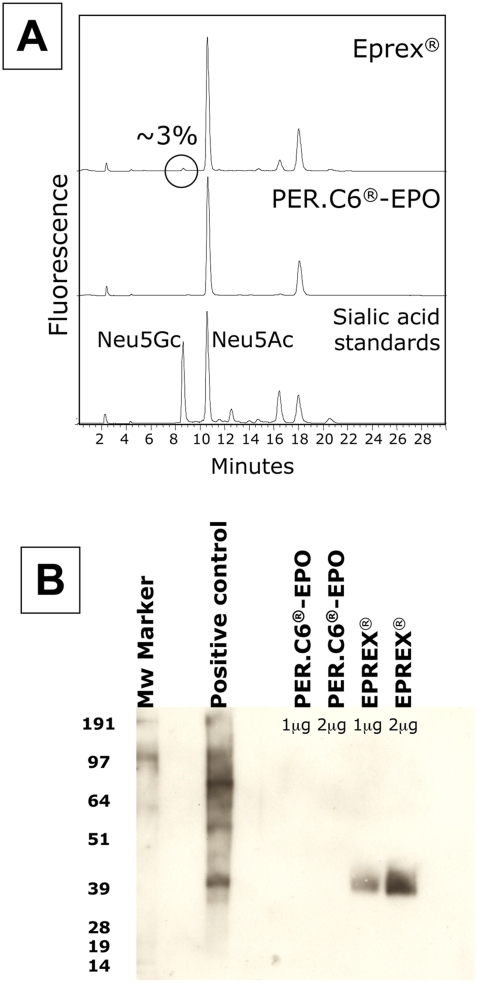
Lack of Neu5Gc in a biotherapeutic agent prepared in the human PER.C6® cell line under Neu5Gc-free/serum-free conditions. PER.C6® cells were stably transfected with cDNA encoding human EPO and co-transfected with ST3GalIV for full sialylation of the glycans of EPO. EPO-expressing PER.C6® cells were cultured under serum-free conditions (VPRO medium) and EPO was purified from the medium. The sialic acid content of PER.C6®-rEPO and of CHO-rEPO (Eprex) was similar as determined by IEF (data not shown). A. The presence of Neu5Gc on CHO-rEPO (Eprex) or PER.C6®-rEPO was examined by DMB-HPLC according to [38]. B. The presence of Neu5Gc on both CHO-rEPO (Eprex) and PER.C6®-rEPO was examined by the highly sensitive Western blot as described in the Methods. 1 and 2 µg of EPO and 5 µg of positive control (bovine fetuin) were run on a 4–12% SDS-PAGE gel and blotted onto nitrocellulose membrane. Immunostaining for Neu5Gc was performed as described in the Methods and as above under figure 2.

As previously reported by us and others, the presence of Neu5Gc on biotherapeutic agents is very likely due to the use of animal cells expressing Neu5Gc and/or the uptake of Neu5Gc from animal derived serum culture media components. To prove this conclusively, we used human cells in defined media to prepare a biotherapeutic agent. PER.C6® cells were generated from retina-derived primary human cells, which were immortalized by insertion of the adenovirus E1 gene [39]. PER.C6® cells are a versatile platform suitable for the production of vectors for gene therapy [40], vaccines [41] and therapeutic glycoproteins such as monoclonal antibodies [42], [43]. As part of further evaluation of PER.C6® cells as a platform for the production of therapeutic glycoproteins these cells were stably transfected with cDNA encoding human erythropoietin (EPO) and co-transfected with a human α2-3sialyltransferase (ST3GalIV) to ensure full sialylation of the glycans of EPO. Transfection, selection and adaptation to serum-free media were performed as described before [42], [43]. The expression plasmid contained the coding sequences for human EPO and human ST3GalIV, both driven by a cytomegalovirus (CMV) promoter that has been modified to achieve high levels of gene expression in PER.C6® cells. An EPO ELISA kit was used to screen for the cell lines with highest expression levels. EPO-expressing PER.C6® cells were cultured under serum-free conditions (VPRO medium) and EPO was purified from the medium by affinity chromatography and ion-exchange chromatography. The sialic acid content of PER.C6®-recombinant erythropoietin (rEPO) and of Eprex (CHO-rEPO, Janssen Cilag) was found to be similar as determined by isoelectric focusing (IEF). The presence of Neu5Gc on both EPO samples was examined by HPLC following 1,2-diamino-4,5-methylene-dioxybenzene (DMB) derivatization, according to [38], and by the highly sensitive Western blot as described in the Methods. In contrast to EPO-derived from CHO cells (Eprex) the PER.C6®-rEPO did not contain sialic acid in the Neu5Gc form, as is expected for a product from a human cell line (Figure 6).

Humans are known to have circulating antibodies against galactoseα1-3-galactose (αGal) and the presence of this epitope on biotherapeutics can cause adverse events [44]. It is now clear that some humans can have similarly high levels of circulating antibodies against Neu5Gc [5]. The use of a human production platform such as PER.C6® cells may avoid problems caused by the presence of non-human glyco-epitopes, since these are absent from biotherapeutics produced in these cells, as shown here by the absence of Neu5Gc on PER.C6®-rEPO.
